# Comparison of Functional Status Improvements Among Patients With Stroke Receiving Postacute Care in Inpatient Rehabilitation vs Skilled Nursing Facilities

**DOI:** 10.1001/jamanetworkopen.2019.16646

**Published:** 2019-12-04

**Authors:** Ickpyo Hong, James S. Goodwin, Timothy A. Reistetter, Yong-Fang Kuo, Trudy Mallinson, Amol Karmarkar, Yu-Li Lin, Kenneth J. Ottenbacher

**Affiliations:** 1 University of Texas Medical Branch, School of Health Professions, Division of Rehabilitation Sciences, Galveston; 2 University of Texas Medical Branch, School of Medicine, Sealy Center on Aging, Department of Internal Medicine, Galveston; 3 University of Texas Health Science Center at San Antonio, School of Health Professions, Department of Occupational Therapy, San Antonio; 4 University of Texas Medical Branch, School of Medicine, Sealy Center on Aging, Department of Preventive Medicine and Population Health, Galveston; 5 George Washington University, School of Medicine and Health Sciences, Clinical Research and Leadership, Washington, DC; 6 University of Texas Medical Branch, School of Health Professions, Division of Rehabilitation Sciences, Galveston; now with Department of Physical Medicine and Rehabilitation, School of Medicine, Virginia Commonwealth University, Richmond; 7 University of Texas Medical Branch, School of Medicine, Department of Preventive Medicine and Population Health, Galveston; 8 University of Texas Medical Branch, School of Health Professions, Sealy Center on Aging, Division of Rehabilitation Sciences, Galveston

## Abstract

**Importance:**

Health care reform legislation and Medicare plans for unified payment for postacute care highlight the need for research examining service delivery and outcomes.

**Objective:**

To compare functional outcomes in patients with stroke after postacute care in inpatient rehabilitation facilities (IRF) vs skilled nursing facilities (SNF).

**Design, Setting, and Participants:**

This cohort study included patients with stroke who were discharged from acute care hospitals to IRF or SNF from January 1, 2013, to November 30, 2014. Medicare claims were used to link to IRF and SNF assessments. Data analyses were conducted from January 17, 2017, through April 25, 2019.

**Exposures:**

Inpatient rehabilitation received in IRFs vs SNFs.

**Main Outcomes and Measures:**

Changes in mobility and self-care measures during an IRF or SNF stay were compared using multivariate analyses, inverse probability weighting with propensity score, and instrumental variable analyses. Mortality between 30 and 365 days after discharge was included as a control outcome as an indicator for unmeasured confounders.

**Results:**

Among 99 185 patients who experienced a stroke between January 1, 2013, and November 30, 2014, 66 082 patients (66.6%) were admitted to IRFs and 33 103 patients (33.4%) were admitted to SNFs. A higher proportion of women were admitted to SNFs (21 466 [64.8%] women) than IRFs (36 462 [55.2%] women) (*P* < .001). Compared with patients admitted to IRFs, patients admitted to SNFs were older (mean [SD] age, 79.4 [7.6] years vs 83.3 [7.8] years; *P* < .001) and had longer hospital length of stay (mean [SD], 4.6 [3.0] days vs 5.9 [4.2] days; *P* < .001) than those admitted to IRFs. In unadjusted analyses, patients with stroke admitted to IRF compared with those admitted to SNF had higher mean scores for mobility on admission (44.2 [95% CI, 44.1-44.3] points vs 40.8 [95% CI, 40.7-40.9] points) and at discharge (55.8 [95% CI, 55.7-55.9] points vs 44.4 [95% CI, 44.3-44.5] points), and for self-care on admission (45.0 [95% CI, 44.9-45.1] points vs 41.8 [95% CI, 41.7-41.9] points) and at discharge (58.6 [95% CI, 58.5-58.7] points vs 45.1 [95% CI, 45.0-45.2] points). Additionally, patients in IRF compared with those in SNF had larger improvements for mobility score (11.6 [95% CI, 11.5-11.7] points vs 3.5 [95% CI, 3.4-3.6] points) and for self-care score (13.6 [95% CI, 13.5-13.7] points vs 3.2 [95% CI, 3.1-3.3] points). Multivariable, propensity score, and instrumental variable analyses showed a similar magnitude of better improvements in patients admitted to IRF vs those admitted to SNF. The differences between SNF and IRF in odds of 30- to 365-day mortality (unadjusted odds ratio, 0.48 [95% CI, 0.46-0.49]) were reduced but not eliminated in multivariable analysis (adjusted odds ratio, 0.72 [95% CI, 0.69-0.74]) and propensity score analysis (adjusted odds ratio, 0.75 [95% CI, 0.72-0.77]). These differences were no longer statistically significant in the instrumental variable analyses.

**Conclusions and Relevance:**

In this cohort study of a large national sample, inpatient rehabilitation in IRFs for patients with stroke was associated with substantially improved physical mobility and self-care function compared with rehabilitation in SNFs. This finding raises questions about the value of any policy that would reimburse IRFs or SNFs at the same standard rate for stroke.

## Key Points

**Question:**

Is change in physical function associated with receiving postacute care after a stroke in inpatient rehabilitation vs skilled nursing facilities?

**Findings:**

This cohort study included 99 185 patients who received postacute care in inpatient rehabilitation or skilled nursing facilities after a stroke. Care in an inpatient rehabilitation facility was associated with greater improvement in mobility and self-care compared with care in a skilled nursing facility, and a significant difference in functional improvement remained after accounting for patient, clinical, and facility characteristics at admission.

**Meaning:**

These findings suggest that there is room for payment reform in postacute care and highlight the need to target decision-making regarding discharge to postacute facilities based on patient needs and potential for recovery.

## Teaser

This cohort study examines the difference in improvement of functional status among patients with stroke who received postacute care in inpatient rehabilitation vs skilled nursing facilities.

## Introduction

More than 40% of Medicare beneficiaries are discharged from acute care hospitals to postacute care each year. Reports by the National Academy of Sciences^1^ and the Institute of Medicine^2^ have found that postacute care was the largest contributor to geographic variation in Medicare costs. The 2014 Improving Medicare Post-Acute Care Transformation (IMPACT) Act^3^ requires the Secretary of the Department of Health and Human Services to establish a unified payment system for postacute care. As a step in this process, the Medicare Payment Advisory Commission recommended that inpatient rehabilitation facilities (IRFs) and skilled nursing facilities (SNFs) explore similar episode-based reimbursement for a given condition. The proposal is based, in part, on the substantial overlap in patient populations served by IRFs and SNFs.^4,5^

The purpose of our study was to examine changes in functional status in a national sample of Medicare beneficiaries with stroke who received inpatient rehabilitation at an IRF or SNF following acute hospital discharge. We selected stroke because it is a major cause of disability in the United States and an important public health issue, patients with stroke have complex neurological disorders that require a range of treatments and expertise, and stroke represents the largest impairment group treated in IRFs.^6^

In this study, we compared functional outcomes of patients with stroke who were discharged from a hospital to an IRF or SNF. There are challenges in comparing outcomes in observational studies, the most important of which is bias by indication, or selection bias. Inpatient rehabilitation facilities have more stringent criteria for admission than do SNFs, including the requirement that patients be able to complete 3 hours of rehabilitation therapy daily. Several studies^7,8,9^ have shown that traditional methods of controlling for patient characteristics, such as logistic regression and propensity analyses, tend not to be effective in the face of strong selection biases. There are several approaches to mitigating this problem. One approach is to assess how large a bias would have to be to eliminate the association observed, which allows the reader to judge whether the existence of such a bias is plausible, such as by use of the E-value.^10^ Another approach is to indirectly assess the strength of the bias and whether it is eliminated by a specific analytic approach, such as by using a control outcome, a measure that should not be affected by differences between the 2 treatments but would be affected by selection biases. In this study, we used all-cause mortality between 30 and 365 days after hospital discharge as a control outcome. The control outcome should be strongly related to the underlying health of the patients but only minimally influenced by residence in an IRF vs SNF. If the statistical analyses show significant IRF vs SNF differences in 30- to 365-day mortality, that result would suggest that underlying selection biases remain. A third approach is to use analytic approaches shown to minimize selection biases, such as instrumental variable analysis.^7,8,9^ We used these 3 approaches to compare outcomes of patients with stroke who were discharged from acute care to IRFs vs SNFs.

We hypothesized that patients discharged to IRFs would have larger improvements in mobility and self-care function than those discharged to SNFs.

## Methods

This study was approved by the institutional review board of the University of Texas Medical Branch and complies with the Centers for Medicare & Medicaid Services (CMS) Data Use Agreement requirements, which waived the need for informed consent for use of the study data because data were deidentified. We reported the study findings according to the Strengthening the Reporting of Observational Studies in Epidemiology (STROBE) reporting guideline.

### Study Data

Our data included Medicare files from 2012 to 2014. These files included Master Beneficiary Summary for patient demographics, Medicare Provider Analysis and Review for claims from hospital and postacute care stays with clinical variables, Inpatient Rehabilitation Facility-Patient Assessment Instrument from IRF,^4,11^ Minimum Data Set 3.0 from SNF,^12^ and the Provider of Services Current Files for hospital characteristics.

### Sample Selection 

The study sample included Medicare beneficiaries 66 years or older discharged from January 1, 2013, to November 30, 2014, to an IRF or SNF after an index acute stay for stroke denoted by Medicare Severity Diagnosis Related Group codes 061 to 066 (eFigure in the Supplement).^13^ Additional inclusion criteria included Medicare Part A coverage without enrollment in a health maintenance organization in the year before and 1 month after the index stroke discharge, residing in the community prior to the index stroke hospitalization, and full mobility and self-care functional measures at the IRF admission and discharge or SNF admission and last follow-up (eTable 1 and eTable 2 in the Supplement).

### Functional Measures: Mobility and Self-Care

Our methods are described in more detail in the eAppendix in the Supplement. We used mobility and self-care items from the Inpatient Rehabilitation Facility-Patient Assessment Instrument and the Minimum Data Set 3.0 (eTable 3 in the Supplement). The Inpatient Rehabilitation Facility-Patient Assessment Instrument includes 5 mobility items and 6 self-care items, with a 7-point rating scale. The Minimum Data Set 3.0 consists of 6 mobility items with a 4-point rating scale and 5 self-care items with a 5-point rating scale.

We used the crosswalk developed by Mallinson et al^14^ to construct comparable admission and discharge functional scores for the postacute care settings.^15^ The scores at admission and discharge for mobility and self-care are reported on a scale of 0 to 100 points, with higher scores indicating greater functional status. This method has demonstrated efficacy in several settings.^16,17^

### Covariates

Patient characteristics included age at admission to IRF or SNF (ie, 66-69, 70-74, 75-79, 80-84, or ≥85 years), sex, race/ethnicity (ie, non-Hispanic white, non-Hispanic black, Hispanic, or other), length of stay (LOS) in acute care (ie, 1-3, 4-7, 8-11, 12-25, or ≥26 days), Medicaid eligibility, type of stroke (ischemic or hemorrhagic) and any stay in intensive care. The race/ethnicity variable was defined by the CMS and was included because some outcomes differ among racial/ethnic groups.^18^ The 30 most frequent CMS Hierarchical Condition Categories for comorbidities were identified through diagnoses on the inpatient claims from the previous year and the secondary diagnoses during the index stroke hospitalization (eTable 4 and eTable 5 in the Supplement).^19^ In addition, we added 6 diagnoses related to cognitive function (eTable 6 in the Supplement). Hospital characteristics included location (urban or rural), hospital type (ie, for-profit, nonprofit, or other), presence of swing beds (yes or no), rehabilitation unit within hospital (yes or no), teaching hospital (yes or no), number of stroke discharges from the index hospital in the same year of the index stroke discharge, and number of beds in index stroke hospital.

### Outcomes

The outcomes were changes in mobility and self-care scores during the IRF or SNF stay. As a control outcome, we assessed mortality between 30 and 365 days after hospital discharge. We selected this outcome to assess how well the analytic techniques controlled for any differences in underlying health status between patients admitted to IRF or SNF. The assumption was that mortality in this time frame would be closely linked to health status and minimally associated with the type of facility.

### Statistical Analysis

Data were analyzed from January 17, 2017, through April 25, 2019. We began with unadjusted bivariate analyses of all variables compared across IRF and SNF settings. We used several analytic approaches to control for potential confounders across IRF and SNF settings, including multivariable analysis, inverse probability weighting with propensity scores and instrumental variable analyses. The multivariable approach used ordinary least squares, adjusting for covariates. Next, we used inverse probability treatment weighting with propensity scores with and without multilevel adjustment.

The propensity score was generated with a logistic regression model using an average treatment effect estimation^20^ that incorporated all covariates listed in eTable 4 and eTable 5 in the Supplement. If any covariates in the propensity score model were not balanced, we additionally controlled for those covariates in the outcome models. Next, we used hierarchical general linear mixed-effects models to account for patients nested within hospitals. Additionally, we used ordinary least squares models with inverse probability treatment weighting, with propensity scores also adjusted for unbalanced covariates, to compare functional status outcome (ie, mobility and self-care) at discharge from IRF or SNF.

We used instrumental variable analysis to adjust for unmeasured confounders across patients and facilities.^21^ The instrumental variables included difference in the distance from the acute care hospital to the nearest IRF vs the nearest SNF, difference in the distance from the beneficiary’s residence to the nearest IRF vs nearest SNF, number of stroke patients discharged to an IRF in the hospital referral region (HRR) in 2013 through 2014, and the previous discharge location assignment (IRF or SNF) for patients with the same type of stroke from the same acute care hospital (eTable 7 and eTable 8 in the Supplement). We estimated the parameters using 2-stage least square regression.^22,23,24^ For the control outcome of 30- to 365-day mortality, the parameters were estimated from 2-stage residual inclusion models because the outcome was dichotomous. Lastly, we calculated E-values for mobility scores, self-care scores, and mortality between patients admitted to IRF or SNF, to assess the potential magnitude of unmeasured confounding that might have produced the results.^10^ Data were analyzed using SAS statistical software version 9.4 (SAS Institute). *P* values were 2-tailed, and statistical significance was set at less than .05.

## Results

A total of 99 185 patients with stroke from 3405 hospitals were included in the study, including 66 082 patients (66.6%) who received stroke rehabilitation in an IRF and 33 103 patients (33.4%) who received stroke rehabilitation in an SNF. Table 1 presents the baseline differences in the patient characteristics between those admitted to IRFs or SNFs. A higher proportion of women were admitted to SNFs (21 466 [64.8%] women) than IRFs (36 462 [55.2%] women) (*P* < .001). Compared with patients admitted to IRFs, patients admitted to SNFs were older (mean [SD] age, 79.4 [7.6] years vs 83.3 [7.8] years; *P* < .001), had longer hospital LOS (mean [SD], 4.6 [3.0] days vs 5.9 [4.2] days; *P* < .001), and had more comorbidities (mean [SD], 2.8 [2.0] comorbidities vs 3.3 [2.1] comorbidities; *P* < .001) (Table 1; eTable 4 in the Supplement). The LOS in SNFs was more than 2-fold that in IRFs (mean [SD], 38.1 [24.1] days vs 15.2 [7.3] days).

**Table 1.** Characteristics of Patients Admission to IRF and SNF

Variable	Patients, No. (%)		*P* Value^a^
	IRF (n = 66 082)	SNF (n = 33 103)	
Age, mean (SD), y^b^	79.4 (7.6)	83.3 (7.8)	<.001
66-69	7959 (12.0)	1869 (5.6)	
70-74	11 994 (18.2)	3244 (9.8)	
75-79	13 421 (20.3)	4931 (14.9)	
80-84	13 931 (21.1)	6978 (21.1)	
≥85	18 777 (28.4)	16 081 (48.6)	
Sex			
Men	29 620 (44.8)	11 637 (35.2)	<.001
Women	36 462 (55.2)	21 466 (64.8)	
Race/ethnicity			
Non-Hispanic white	52 826 (79.9)	26 775 (80.9)	<.001
Non-Hispanic black	7753 (11.7)	3915 (11.9)	
Hispanic	3202 (4.9)	1371 (4.1)	
Other	2301 (3.5)	1042 (3.1)	
Stroke type			
Ischemic	58 872 (89.1)	29 272 (88.4)	.002
Hemorrhagic	7210 (10.9)	3831 (11.6)	
Length of stay in acute care, mean (SD), d^b^	4.6 (3.0)	5.9 (4.2)	<.001
1-3	28 099 (42.5)	9723 (29.4)	
4-7	29 996 (45.4)	16 403 (49.6)	
8-11	5839 (8.8)	4390 (13.3)	
12-25	2066 (3.1)	2403 (7.3)	
≥26	82 (0.1)	184 (0.6)	
Admission function score, mean (SD)^c^			
Mobility^d^	44.2 (7.4)	40.8 (9.4)	<.001
Self-care^e^	45.0 (11.1)	41.9 (11.7)	<.001
No. of comorbidities, mean (SD)^b^	2.8 (2.0)	3.3 (2.1)	<.001
Medicaid eligible	10 454 (15.8)	7222 (21.8)	<.001
Stayed in ICU or CCU	39 195 (59.3)	17 178 (51.9)	<.001
Urban hospital	60 114 (91.0)	28 207 (85.2)	<.001
Hospital type			
For-profit	9480 (14.3)	4074 (12.3)	<.001
Nonprofit	48815 (73.9)	24 848 (75.1)	
Other	7787 (11.8)	4181 (12.6)	
Swing bed	1710 (2.6)	2023 (6.1)	<.001
Rehabilitation unit in IRF^f^	40 742 (61.7)	14 657 (44.3)	<.001
Teaching hospital	34 919 (52.8)	15 858 (47.9)	<.001
Stroke discharges, No., mean (SD)^b^	248.0 (175.9)	218.7 (174.8)	<.001
Hospital beds, No., mean (SD)^b^	463.0 (329.2)	414.2 (332.0)	<.001

Abbreviations: CCU, cardiac care unit; ICU, intensive care unit; IRF, inpatient rehabilitation facilities; SNF, skilled nursing facilities.

^a^ Based on χ^2^ test.

^b^ Based on Wilcoxon rank sum test.

^c^ Scores were scaled on 0- to 100-point scales, with higher scores indicating greater functional status.

^d^ Mobility score for IRF measured the level of help needed for transfer to bed, chair, or wheelchair, transfer to toilet, transfer tub or shower, locomotion via walking or a wheelchair, and locomotion on stairs. Mobility score for SNF measured the level of help needed for bed mobility, transfer, walking in a room, walking in a corridor, locomotion on the unit, and locomotion off the unit.

^e^ Self-care scores in IRF measured the level of help needed for eating, grooming, bathing, dressing upper body, dressing lower body, and toileting. For SNF, self-care score measured the level of help needed for dressing, eating, toilet use, personal hygiene, and bathing.

^f^ Indicates a rehabilitation unit that is part of an acute care hospital rather than a free-standing rehabilitation facility.

Table 2 presents the unadjusted mobility and self-care scores at admission and discharge for patients in IRFs and SNFs, along with the change in scores between admission and discharge. Compared with patients in IRFs, patients in SNFs had lower mean scores for mobility (44.2 [95% CI, 44.1-44.3] points vs 40.8 [95% CI, 40.7-40.9] points) and self-care (45.0 [95% CI, 44.9-45.1] points vs 41.8 [95% CI, 41.7-41.9] points) at admission and for mobility (55.8 [95% CI, 55.7-55.9] points vs 44.4 [95% CI, 44.3-44.5] points) and self-care (58.6 [95% CI, 58.5-58.7] points vs 45.1 [95% CI, 45.0-45.2] points) at discharge. The changes in mobility and self-care scores were substantially greater among IRF patients. For mobility, the change was 11.6 (95% CI, 11.5-11.7) points for patients in IRFs vs 3.5 (95% CI, 3.4-3.6) points for those in SNFs. For self-care, the change was 13.6 (95% CI, 13.5-13.7) points vs 3.2 (95% CI, 3.1-3.3) points.

**Table 2.** Unadjusted Admission and Discharge Results

Score	Mean (95% CI)			
	IRF		SNF	
	Mobility	Self-care	Mobility	Self-care
At admission	44.2 (44.1-44.3)	45.0 (44.9-45.1)	40.8 (40.7-40.9)	41.8 (41.7-41.9)
At discharge	55.8 (55.7-55.9)	58.6 (58.5-58.7)	44.4 (44.3-44.5)	45.1 (45.0-45.2)
Change	11.6 (11.5-11.7)	13.6 (13.5-13.7)	3.5 (3.4-3.6)	3.2 (3.1-3.3)

Abbreviations: IRF, inpatient rehabilitation facilities; SNF, skilled nursing facilities.

After applying propensity score weights, most demographics and comorbidities were balanced between IRF and SNF (49 of 52 variables [94.2%]) (eTable 4 and eTable 5 in the Supplement). Table 3 presents stroke outcomes by mobility and self-care discharge scores for patients in IRF or SNF. Regardless of covariate adjustment method, the patients with stroke who were discharged from IRF had higher mobility and self-care scores than those discharged from SNF. In multivariate adjustment analysis, the mean (SE) difference in scores between patients from IRF vs SNF was 7.8 (0.05) points for mobility and 9.7 (0.06) points for self-care. In the multilevel multivariate propensity score inverse probability of treatment weighting model, the mean (SE) difference in scores between patients from IRF vs SNF was 8.0 (0.04) points for mobility and 9.9 (0.05) points for self-care. Results of instrumental variable analyses are summarized in Table 3 and show similar results, including by differential distance from acute care hospital to nearest IRF or SNF (mean [SE] difference: mobility score, 8.2 [0.34] points; self-care score, 9.8 [0.39] points), by differential distance from patient’s residence to nearest IRF or SNF (mean [SE] difference: mobility score, 5.6 [0.63] points; self-care score, 8.7 [0.72] points), by percentage of IRFs within the acute hospital HRR (mean [SE] difference: mobility score, 10.4 [0.21] points; self-care score, 11.9 [0.25] points), and by previous IRF or SNF assignment by stroke type within each hospital (mean [SE] difference: mobility score, 9.2 [0.30] points; self-care score, 10.7 [0.34] points). In all models, the changes in mobility and self-care scores for those discharged from IRFs were at least 2-fold those for patients discharged from SNFs.

**Table 3.** Change in Score From Admission to Discharge in IRF and SNF

Analysis	Score, Mean (SE)					
	IRF		SNF		Difference	
	Mobility	Self-care	Mobility	Self-care	Mobility	Self-care
Estimation method						
Unadjusted	11.6 (0.03)	13.6 (0.04)	3.5 (0.03)	3.2 (0.04)	8.0 (0.05)	10.4 (0.06)
Multivariate adjustment	11.5 (0.03)	13.4 (0.03)	3.7 (0.04)	3.7 (0.05)	7.8 (0.05)	9.7 (0.06)
Propensity score models						
Multivariate IPTW adjustment^a^	11.5 (0.03)	13.4 (0.03)	3.5 (0.03)	3.4 (0.03)	8.0 (0.04)	9.9 (0.05)
Multilevel multivariate IPTW adjustment	11.4 (0.03)	13.2 (0.04)	3.4 (0.03)	3.4 (0.04)	8.0 (0.04)	9.9 (0.05)
Instrumental variable analysis						
Differential distance from acute to nearest IRF or SNF	11.7 (0.12)	13.4 (0.13)	3.4 (0.23)	3.6 (0.26)	8.2 (0.34)	9.8 (0.39)
Differential distance from beneficiary to nearest IRF or SNF	10.8 (0.21)	13.1 (0.24)	5.2 (0.42)	4.4 (0.48)	5.6 (0.63)	8.7 (0.72)
Percentage of IRFs within acute hospital referral region	12.4 (0.07)	14.2 (0.09)	2.0 (0.14)	2.2 (0.16)	10.4 (0.21)	11.9 (0.25)
Previous IRF or SNF assignment by stroke type within each hospital	12.0 (0.10)	13.7 (0.12)	2.8 (0.20)	3.0 (0.23)	9.2 (0.30)	10.7 (0.34)

Abbreviations: IPTW, inverse probability of treatment weighting; IRF, inpatient rehabilitation facility; SNF, skilled nursing facility.

^a^ After applying propensity score weights, most demographics and stroke comorbidities were balanced between IRF and SNF (49 out of 52 variables), except for admission mobility score (IRF mean [SD], 43.3 [6.6]; SNF, 43.7 [12.0]; *P* < .001), admission self-care score (IRF, 44.0 [9.8]; SNF, 44.3 [14.3]; *P* = .001), and hemiplegia or hemiparesis (IRF, 43.7%; SNF, 42.7%; *P* = .02).

In order to assess the ability of the various analytic techniques to adjust for unmeasured confounders, we assessed mortality between 30 and 365 days as a control outcome (Table 4). In unadjusted analyses, patients with stroke who were discharged from IRF had lower mortality than those discharged from SNF (17.5% vs 30.5%, OR, 0.48 [95% CI, 0.46-0.49]). Adjustment for patient and hospital characteristics in a multivariate adjustment model increased the OR to 0.72 (95% CI, 0.69-0.74), which was similar to results of the inverse probability weighted propensity models (adjusted odds ratio, 0.75 [95% CI, 0.72-0.77]). In contrast, the 4 instrumental variable models resulted in odds of mortality closer to 1.0, with ORs ranging from 0.92 (95% CI, 0.76-1.11) when adjusted for previous IRF or SNF assignment by stroke type within each hospital to 1.25 (95% CI, 0.88-1.76) when adjusted by differential distance from patient’s residence to the nearest IRF or SNF (Table 4).

**Table 4.** 30- to 365-d Mortality From Hospital Discharge Between IRFs and SNFs

Analysis	Odds Ratio (95% CI)
Estimation method	
Unadjusted	0.48 (0.46-0.49)
Multivariate adjustment	0.72 (0.69-0.74)
Propensity score model	
Multivariate IPTW adjustment	0.75 (0.72-0.77)
Multilevel multivariate IPTW adjustment	0.72 (0.69-0.74)
Instrumental variable	
Differential distance from acute to nearest IRF or SNF	1.01 (0.82-1.23)
Differential distance from beneficiary to nearest IRF or SNF	1.25 (0.88-1.76)
Percentage of IRFs with the acute hospital referral region	1.02 (0.89-1.17)
Previous IRF or SNF assignment by stroke type within each hospital	0.92 (0.76-1.11)

Abbreviations: IPTW, inverse probability of treatment weighting; IRF, inpatient rehabilitation facilities; SNF, skilled nursing facilities.

Lastly, for each outcome, we calculated the E-value to assess the minimum strength of association that an unmeasured confounder would need to have with the outcome and postacute care setting to eliminiate the association between postacute care setting and each outcome (eTable 9 in the Supplement). The lower confidence limit of the E-value was 4.0 for the change in mobility and 4.2 for self-care scores. E-values this large indicate that the association between function score change and postacute care setting we observed was strong.^10^

## Discussion

Currently, the decision-making process in selecting postacute care services is heavily influenced by nonclinical factors.^25,26,27,28,29,30^ This is shown by the substantial geographic variation in the proportions of patients with stroke discharged to IRFs or SNFs.^28^ The choice is associated with measures of availability, such as distance to the nearest facility.^29^ The association of IRF vs SNF use with these nonclinical factors allows investigators to use them as instruments in an instrumental variable analysis, which should better control for unmeasured confounders that might be influencing the choice of IRF vs SNF.

Comparative research related to functional outcomes for persons with stroke receiving rehabilitation in IRFs vs SNFs is limited, to our knowledge. A recent systematic review reported better functional outcomes and higher costs for patients in IRFs compared with those in SNFs and emphasized the need for additional research.^4^ Limited research has reported generally better functional outcomes associated with patients in IRFs vs SNFs after a stroke.^4,29,31,32^ The findings of our study support this trend. In the 4 instrumental variable models, the differences in improvement in mobility scores between IRF and SNF patients between 5 and 10 points and for self-care scores, the difference was between 8 and 12 points. A 10-point difference in self-care in an IRF is the difference between a patient rating of needing maximal assistance vs needing supervision. Maximal assistance requires another person to physically assist the patient. Needing supervision simply involves another person being present to monitor the activity but not provide physical assistance unless required. Patients at the level of needing supervision are usually ready for discharge to home, while patients needing maximal assistance will require continued institutional care or in-home nursing support after discharge from postacute care.^32,33^

We also found differences in functional outcomes between IRF and SNF using logistic regression and propensity scores. However, the inability of more analytical techniques to eliminate the differences in the control outcome of all-cause mortality between 30 and 365 days suggests that those approaches did not eliminate selection biases. This pattern is consistent with prior comparative effectiveness studies using observational data^7,8,9^ and reinforces the view that such techniques should be avoided in the face of strong selection bias.

Our study adds to the accumulating scientific literature that better functional outcomes, such as mobility and self-care, are associated with discharge from IRFs vs SNFs among stroke survivors.^4,29,31,32^ This has not been true for other conditions, such as hip fracture or joint replacement.^34^ A study by Mallinson et al^34^ comparing mobility and self-care outcomes, which were measured in the same way as in our study, among patients with hip fracture receiving rehabilitation from IRFs, SNFs, or home health agencies found no statistically significant differences in fully adjusted models. The difference in findings between the Mallinson et al study^34^ and our study could be related to many factors. We believe the difference in conditions (ie, hip fracture and joint replacement vs stroke) is the most plausible explanation.

Stroke is a complex neurological condition affecting multiple body systems and requiring intensive rehabilitation from several disciplines with different areas of expertise. An IRF is designed to provide intensive rehabilitation to complex patients who need specialized care. To effectively and safely implement unified payment in postacute care,^3^ it will be necessary to recognize differences in the rehabilitation needs of patients with stroke and other complex conditions. The CMS 60% rule identifies 13 diagnostic conditions that classify a facility as an IRF for Medicare reimbursement.^35^ Stroke is the largest category of these conditions, with 20.5% of all patients in IRFs in 2017.^6^

The instrumental variable analyses in this study describe the outcomes of the marginal patient, that is, those patients who reasonably could have been discharged either to an IRF or SNF. The assumption is that there are patients at the ends of the spectrum who are highly likely to be discharged to an IRF or SNF, but that there are also patients in the middle who could go to either one and for whom the choice is influenced by nonclinical factors. It is not possible to directly measure the size of the population of marginal patients. In a study of Medicare spending and outcomes after postacute care for stroke and hip fracture, Buntin et al^36^ estimated the percentage or marginal patients as between 20% to 30% of patients with hip fracture or stroke. One way to estimate the size of the marginal patient population is to examine the distribution in variation in percentage of patients with stroke discharged to an IRF or SNF among HRRs. The assumption is that the underlying health of patients with stroke would vary somewhat among HRRs, but not markedly, and that the variation reflects local availability of the 2 types of facilities along with other medical cultural issues. Our findings are similar to what Buntin et al^36^ estimated as the percentage of patients with marginal stroke and hip fracture. Our findings and the research of Buntin et al^36^ indicate that it may be possible to improve our ability to identify appropriate candidates for the high-intensity, specialized services provided in IRFs.

Additional research is necessary to confirm our findings and to identify whether any of the other 13 conditions identified by CMS as priority diagnoses for receiving services in IRFs (the 60% rule) may also show differences in functional outcomes based on treatment in IRFs vs SNFs. Our findings also have implications regarding the IMPACT Act.^3^ Studies that compare functional outcomes for all patients discharged to postacute care may be missing treatment effects that appear only in some impairment groups requiring the intense or specialized rehabilitation available in IRFs.^30^ For many hospital discharges, the postacute care setting may not matter, but our results suggest that, for at least one-third of patients with a stroke, discharge to an IRF vs SNF was associated with a significant difference in self-care and mobility at discharge.

As the IMPACT Act^3^ and unified payment are implemented, it will be important to accurately identify subgroups and target patients who would do better in one setting vs another. The current CMS rules for identifying priority patients for IRFs are a good start, but challenges remain, such as the large disparity in the availability of IRFs vs SNFs. Another concern is the current cost differential between postacute care settings. The Medicare Payment Advisory Commission reports^6,37^ consistently demonstrate that IRF costs are higher than those of SNF and home health. In a unified payment system, there would be financial incentives to shift high-cost patients, such as patients with stroke and other complex medical conditions, to lower-cost postacute care options. Effective administrative oversight will be required to ensure patients receive the appropriate care in the right setting.

### Limitations

This study has limitations. Our findings are based on Medicare files for IRF and SNF settings only and are not applicable to stroke rehabilitation in other postacute venues (eg, home health care, long-term care hospitals, or outpatient care). We were not able to examine cognitive function before and after the stroke, stroke severity, or location of the stroke. The number of items to measure cognitive function in the IRF and SNF assessment protocols are small, and our preliminary analyses to develop a cocalibrated crosswalk revealed low precision.^16,38^ Instead, we included diagnoses associated with cognitive dysfunction in the comorbidities that were controlled for (eTable 6 in the Supplement). The development of a standardized measure of cognitive function is an important area for future research and is included as part of the IMPACT Act.^3^ Previous investigations have consistently reported that the costs for rehabilitation services provided in SNFs are significantly lower than in IRFs, even when the longer LOSs associated with SNFs are considered.^4,36^ We did not conduct cost comparisons or cost benefit analyses associated with outcomes across the 2 postacute settings. This is an important topic for future research.

## Conclusions

This cohort study found that Medicare beneficiaries who received services at an IRF after a stroke demonstrated greater improvement in mobility and self-care compared with patients who received inpatient rehabilitation at a SNF. A significant difference in functional improvement remained after accounting for patient, clinical, and facility characteristics at admission. Our findings indicate the need to carefully manage discharge to postacute care based on the patient’s needs and potential for recovery. Postacute care reform based on the IMPACT Act^3^ must avoid a payment system that shifts patients with stroke who could benefit from intensive inpatient rehabilitation to lower cost settings.

## Notes

Supplement. **eFigure.** Study Flow Diagram for the Sample **eTable 1.** Patient Characteristics Between Skilled Nursing Facility (SNF) Stays Included in the Cohort and Those Excluded Owing to Incomplete Data for Function Scores **eTable 2.** Patient Comorbidities Between Skilled Nursing Facility (SNF) Stays Included in the Cohort and Those Excluded Due to Incomplete Data for Function Scores **eAppendix.** eMethods **eTable 3.** Comparison of Inpatient Rehabilitation Facility-Patient Assessment Instrument (IRF-PAI) With Minimum Data Set 3.0 (MDS) Items in the Mobility and Self-care Construct **eTable 4.** Demographics Across Inpatient Rehabilitation Facilities (IRFs) and Skilled Nursing Facilities (SNFs) Before and After Inverse Probability of Treatment Weighting **eTable 5.** Stroke Comorbidities Across Inpatient Rehabilitation Facilities (IRFs) and Skilled Nursing Facilities (SNFs) Before and After Inverse Probability of Treatment Weighting **eTable 6.** Additional Diagnoses Related to Cognitive Function **eTable 7.** Instrumental Variables Across Inpatient Rehabilitation Facilities (IRFs) and Skilled Nursing Facilities (SNFs) **eTable 8.** Standardized Difference for Instrumental Variables **eTable 9.** E-values for Mobility and Self-care Scores and 30- to 365-Day Mortality From Hospital Discharge **eReferences**
