# Supplementary material for: Comparison of Functional Status Improvements Among Patients With Stroke Receiving Postacute Care in Inpatient Rehabilitation vs Skilled Nursing Facilities
Source: JAMA Netw Open. 2019 Dec 4;2(12):e1916646. doi: 10.1001/jamanetworkopen.2019.16646 (PMC6902754; doi:10.1001/jamanetworkopen.2019.16646)
Supplement: Supplement. — eFigure. Study Flow Diagram for the Sample eTable 1. Patient Characteristics Between Skilled Nursing Facility (SNF) Stays Included in the Cohort and Those Excluded Owing to Incomplete Data for Function Scores eTable 2. Patient Comorbidities Between Skilled Nursing Facility (SNF) Stays Included in the Cohort and Those Excluded Due to Incomplete Data for Function Scores eAppendix. eMethods eTable 3. Comparison of Inpatient Rehabilitation Facility-Patient Assessment Instrument (IRF-PAI) With Minimum Data Set 3.0 (MDS) Items in the Mobility and Self-care Construct eTable 4. Demographics Across Inpatient Rehabilitation Facilities (IRFs) and Skilled Nursing Facilities (SNFs) Before and After Inverse Probability of Treatment Weighting eTable 5. Stroke Comorbidities Across Inpatient Rehabilitation Facilities (IRFs) and Skilled Nursing Facilities (SNFs) Before and After Inverse Probability of Treatment Weighting eTable 6. Additional Diagnoses Related to Cognitive Function eTable 7. Instrumental Variables Across Inpatient Rehabilitation Facilities (IRFs) and Skilled Nursing Facilities (SNFs) eTable 8. Standardized Difference for Instrumental Variables eTable 9. E-values for Mobility and Self-care Scores and 30- to 365-Day Mortality From Hospital Discharge eReferences [file jamanetwopen-2-e1916646-s001.pdf]

Supplementary Online Content

Hong I, Goodwin JS, Reistetter TA, et al. Comparison of functional status improvements among patients with stroke receiving postacute care in inpatient rehabilitation vs skilled nursing facilities. *JAMA Netw Open.* 2019;2(12):e1916646. doi:10.1001/jamanetworkopen.2019.16646

eFigure. Study Flow Diagram for the Sample

eTable 1. Patient Characteristics Between Skilled Nursing Facility (SNF) Stays Included in the Cohort and Those Excluded Owing to Incomplete Data for Function Scores

eTable 2. Patient Comorbidities Between Skilled Nursing Facility (SNF) Stays Included in the Cohort and Those Excluded Due to Incomplete Data for Function Scores

eAppendix. eMethods

eTable 3. Comparison of Inpatient Rehabilitation Facility-Patient Assessment Instrument (IRF-PAI) With Minimum Data Set 3.0 (MDS) Items in the Mobility and Self-care Construct

eTable 4. Demographics Across Inpatient Rehabilitation Facilities (IRFs) and Skilled Nursing Facilities (SNFs) Before and After Inverse Probability of Treatment Weighting

eTable 5. Stroke Comorbidities Across Inpatient Rehabilitation Facilities (IRFs) and Skilled Nursing Facilities (SNFs) Before and After Inverse Probability of Treatment Weighting

eTable 6. Additional Diagnoses Related to Cognitive Function

eTable 7. Instrumental Variables Across Inpatient Rehabilitation Facilities (IRFs) and Skilled Nursing Facilities (SNFs)

eTable 8. Standardized Difference for Instrumental Variables

eTable 9. E-values for Mobility and Self-care Scores and 30- to 365-Day Mortality From Hospital Discharge

eReferences

This supplementary material has been provided by the authors to give readers additional information about their work.

© 2019 Hong I et al. *JAMA Network Open.*

eFigure. Study Flow Diagram for the Sample

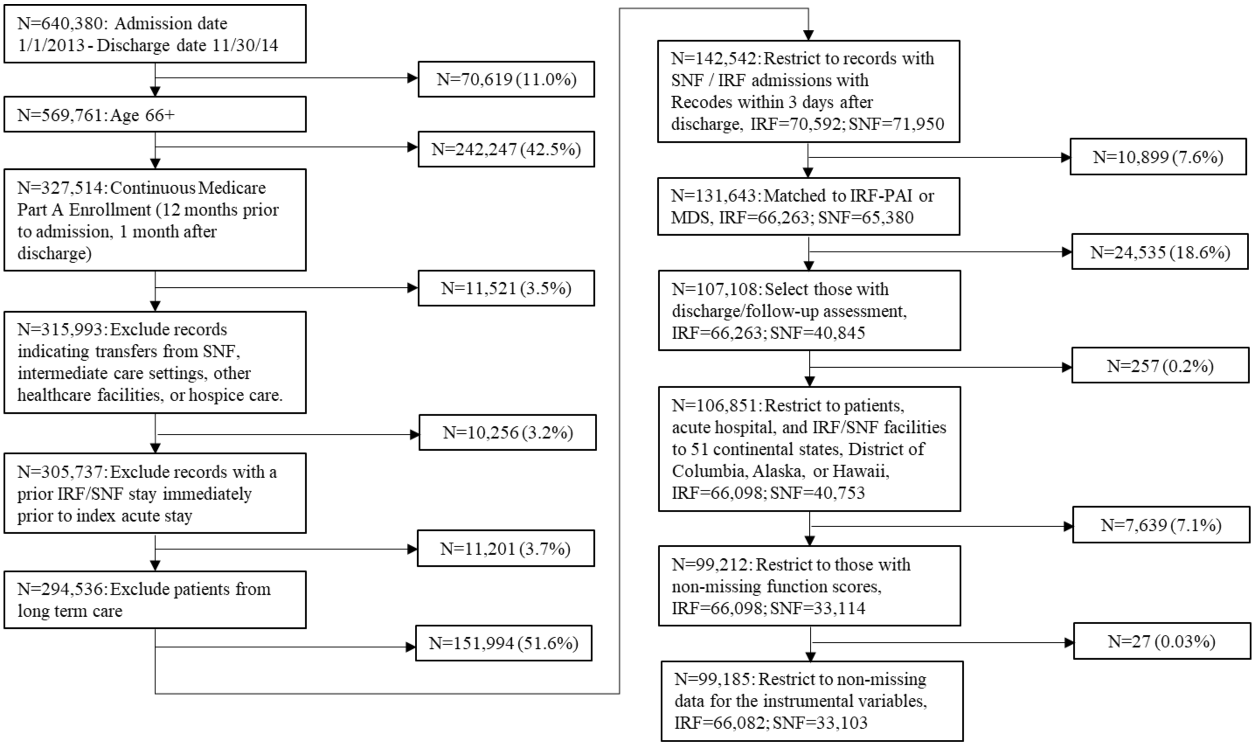

© 2019 Hong I et al. *JAMA Network Open.*

eTable 1. Patient Characteristics Between Skilled Nursing Facility (SNF) Stays Included in the Cohort and Those Excluded Owing to Incomplete Data for Function Scores

Patient characteristics	Included (N=33103), No. (%)	Excluded (N=7639), No. (%)	p†	Standardized Difference
**Age (years)**				0.0764
66-69	1869 (5.65)	342 (4.48)	< .0001*	
70-74	3244 (9.80)	727 (9.52)		
75-79	4931 (14.90)	1052 (13.77)		
80-84	6978 (21.08)	1565 (20.49)		
85+	16081 (48.58)	3953 (51.75)		
**Sex**				0.0196
Male	11637 (35.15)	2614 (34.22)	0.1225	
Female	21466 (64.85)	5025 (65.78)		
**Race**				0.0808
Non-Hispanic White	26775 (80.88)	6411 (83.92)	< .0001*	
Non-Hispanic Black	3915 (11.83)	776 (10.16)		
Hispanic	1371 (4.14)	254 (3.33)		
Other race	1042 (3.15)	198 (2.59)		
**Stroke type**				-0.0142
Ischemic	29272 (88.43)	6720 (87.97)	0.2615	
Hemorrhagic	3831 (11.57)	919 (12.03)		
**Length of stay in acute care**	5.9 (4.2)	5.9 (4.1)	0.5563	-0.0084
**Length of stay in SNF**	38.1 (24.1)	39.1 (23.8)	< .0001*	0.0422
**Medicaid eligibility (Yes)**	7222 (21.82)	1473 (19.28)	< .0001*	-0.0627
**Stay in ICU/CCU (Yes)**	17178 (51.89)	4106 (53.75)	0.0034*	0.0372
**Urban hospital (Yes)**	28207 (85.21)	6348 (83.10)	< .0001*	-0.0578
**Hospital control type**				0.0457
Profit	4074 (12.31)	855 (11.19)	0.0015*	
Non Profit	24848 (75.06)	5730 (75.01)		
Other	4181 (12.63)	1054 (13.80)		
**Swing bed (Yes)**	2023 (6.11)	458 (6.00)	0.7031	-0.0049
**Rehabilitation unit in IRF (Yes)**	14657 (44.28)	3605 (47.19)	< .0001*	0.0585
**Teaching hospital (Yes)**	15858 (47.91)	3741 (48.97)	0.0924	0.0214
**Number of stroke discharges, Mean (SD)**	218.7 (174.9)	223.8 (180.4)	0.1776	0.0283
**Number of beds in the hospital, Mean (SD)**	414.2 (332.0)	412.5 (337.8)	0.0703	-0.0051

†, Wilcoxon Rank Sum test for length of stay in acute care and SNF, number of stroke discharges and number of beds in the hospital; Chi-square test for others.
*, Statistically significant at a significance level of 0.05

© 2019 Hong I et al. *JAMA Network Open.*

eTable 2. Patient Comorbidities Between Skilled Nursing Facility (SNF) Stays Included in the Cohort and Those Excluded Due to Incomplete Data for Function Scores

**Comorbidity**	**Included (N=33103), No. (%)**	**Excluded (N=7639), No. (%)**	**p†**	**Standardized Difference**
Hemiplegia/Hemiparesis	12154 (36.72)	2753 (36.04)	0.2682	-0.0141
Specified Heart Arrhythmias	13133 (39.67)	3063 (40.10)	0.4952	0.0087
Diabetes without Complication	9169 (27.70)	1958 (25.63)	0.0003*	-0.0468
Congestive Heart Failure	8918 (26.94)	2160 (28.28)	0.0180*	0.0299
Acute Renal Failure	5833 (17.62)	1423 (18.63)	0.0381*	0.0262
Chronic Obstructive Pulmonary Disease	5317 (16.06)	1243 (16.27)	0.6530	0.0057
Vascular Disease	4242 (12.81)	1025 (13.42)	0.1565	0.0179
Ischemic or Unspecified Stroke	2807 (8.48)	638 (8.35)	0.7176	-0.0046
Diabetes with Chronic Complications	2388 (7.21)	577 (7.55)	0.3032	0.0130
Coagulation Defects and Other Specified Hematological Disorders	2196 (6.63)	523 (6.85)	0.5021	0.0085
Seizure Disorders and Convulsions	2383 (7.20)	546 (7.15)	0.8759	-0.0020
Coma, Brain Compression/Anoxic Damage	1851 (5.59)	444 (5.81)	0.4509	0.0095
Cardio-Respiratory Failure and Shock	2016 (6.09)	543 (7.11)	0.0009*	0.0410
Cerebral Hemorrhage	1600 (4.83)	369 (4.83)	0.9914	-0.0001
Protein-Calorie Malnutrition	2176 (6.57)	589 (7.71)	0.0004*	0.0442
Rheumatoid Arthritis and Inflammatory Connective Tissue Disease	1392 (4.21)	321 (4.20)	0.9908	-0.0001
Morbid Obesity	1141 (3.45)	292 (3.82)	0.1081	0.0201
Aspiration and Specified Bacterial Pneumonias	1536 (4.64)	438 (5.73)	< .0001*	0.0493
Acute Myocardial Infarction	1464 (4.42)	346 (4.53)	0.6829	0.0052
Other Significant Endocrine and Metabolic Disorders	1199 (3.62)	281 (3.68)	0.8121	0.0030
Septicemia, Sepsis, Systemic Inflammatory Response Syndrome/Shock	1288 (3.89)	310 (4.06)	0.4973	0.0086
Parkinson's and Huntington's Diseases	1025 (3.10)	256 (3.35)	0.2500	0.0144
Breast, Prostate, and Other Cancers and Tumors	856 (2.59)	195 (2.55)	0.8690	-0.0021
Major Depressive, Bipolar, and Paranoid Disorders	800 (2.42)	193 (2.53)	0.5748	0.0071
Vascular Disease with Complications	582 (1.76)	109 (1.43)	0.0433*	-0.0265
Unstable Angina and Other Acute Ischemic Heart Disease	536 (1.62)	136 (1.78)	0.3189	0.0125
Intestinal Obstruction/Perforation	585 (1.77)	130 (1.70)	0.6947	-0.0050
Hip Fracture/Dislocation	662 (2.00)	171 (2.24)	0.1839	0.0166
Metastatic Cancer and Acute Leukemia	457 (1.38)	107 (1.40)	0.8918	0.0017
Vertebral Fractures without Spinal Cords Injury	549 (1.66)	165 (2.16)	0.0026*	0.0367
**Cognitive function related variables**				

© 2019 Hong I et al. *JAMA Network Open.*

Dementia without Complication	9910 (29.94)	2397 (31.38)	0.0134*	0.0313
Delirium and Encephalopathy	5736 (17.33)	1421 (18.60)	0.0083*	0.0332
Nonpsychotic Organic Brain Syndromes/Conditions	1322 (3.99)	307 (4.02)	0.9191	0.0013
Dementia with Complications	1363 (4.12)	353 (4.62)	0.0483*	0.0246
Concussion/Head Injury	696 (2.10)	163 (2.13)	0.8639	0.0022
Schizophrenia	187 (0.56)	27 (0.35)	0.0212*	-0.0313

†, Chi-square test.
*, Statistically significant at a significance level of 0.05

© 2019 Hong I et al. *JAMA Network Open.*

eAppendix. eMethods

Sample Selection

The study sample included Medicare beneficiaries age ≥66 years discharged from January 1, 2013 to November 30, 2014 to an inpatient rehabilitation facility (IRF) or skilled nursing facility (SNF) following an index acute stay for stroke, denoted by Medicare Severity Diagnosis Related Group codes 061-066. Additional inclusion criteria included 1) Medicare Part A coverage without enrollment in a Health Maintenance Organization in the year before and 1 month after the index stroke discharge, 2) residing in the community prior to the index stroke hospitalization and 3) with full mobility and self-care functional measures at the IRF admission and discharge or SNF admission and last follow-up (eFigure 1). In this cohort section, we removed 7,639 observations due to incomplete data for function scores in SNF. To check for differences in the baseline variables between those who remained in the analysis and those excluded because of missing outcome data, we compared the original sample and the sub-cohort which excluded the 7.1% with incomplete data for function scores in SNFs. Even though some of the Chi-square p-values are significant, none of the absolute standardized differences were > 0.1. This means these two groups are balanced. We included this information in the supplementary materials (eTable 1 and eTable 2).

Functional Measures: Mobility and Self-care

Post-acute care assessment data were used to characterize both stroke severity as well as outcomes of care using admission and discharge functional measures. The Centers for Medicare and Medicaid Services (CMS) documentation requirements for the Inpatient Rehabilitation Facility-Patient Assessment Instrument[1] and the Minimum Data Set 3.0[2] both contain functional information used to document changes in patient abilities from admission to discharge (in the Inpatient Rehabilitation Facility-Patient Assessment Instrument) or from admission to follow-up assessments (in the Minimum Data Set 3.0). In this study, we utilized only the mobility and self-care test items from these instruments (eTable 3). The Inpatient Rehabilitation Facility-Patient Assessment Instrument has five mobility items: *bed/chair/wheelchair transfer, toilet transfer, tub/shower transfer, locomotion-walk/wheelchair,* and *locomotion-stairs*; and six self-care items: *eating, grooming, bathing, dressing-upper extremity, dressing-lower extremity,* and *toileting,* scored with a 7-point rating scale (1-total assistant to 7-complete independence). Similarly, the Minimum Data Set consists of six mobility items: including *bed mobility, transfer, walk in room, walk in corridor, locomotion on unit*, and *locomotion off unit*; and five self-care items: *eating, personal hygiene, bathing, dressing,* and *toilet use*. The Minimum Data Set 3.0 has two different rating scales: a 5-point self-performance rating scale (0-independnet to 4-total dependence) and a 4-point support rating scale (0-no to 3-more than two persons' physical assistance).

Using data from the Inpatient Rehabilitation Facility-Patient Assessment Instrument and the Minimum Data Set 3.0, we used the crosswalk developed by Mallinson and colleagues (2011) to construct comparable admission and discharge functional scores for the two post-acute care settings.[3] The co-calibrated crosswalk contains mobility and self-care domains and was established using a Rasch common-person equating method.[4] This measurement method has demonstrated efficacy for equating similar activities of daily living instruments.[5,6] For instance, crosswalks have been successfully developed between the Functional Independence Measure and the Minimum Data Set for veteran patients, and between the Functional Independence Measure and the modified Barthel Index for Korean adult patients. These crosswalks also demonstrated good psychometric properties. The co-calibrated functional scores at admission and discharge for mobility and self-care were reported on a 0-100 point scale, where higher scores indicate greater functional status, which is consistent with the Rasch measurement method.

Covariates – Hierarchical Condition Categories

We used CMS Hierarchical Condition Categories for comorbidities identified through diagnoses on the inpatient claims from the previous year and the secondary diagnoses during the index stroke hospitalization. While the primary purpose of CMS Hierarchical Condition Categories is for adjusting risk in the Medicare Advantage payment plans, it can also be used to risk-adjust patient-level health conditions.[7] Among the 79 categories, we used the 30 most frequent CMS Hierarchical Condition Categories (yes, no) in the analysis.[8]

Statistical analysis

We began with unadjusted bivariate analyses of all variables compared across IRF and SNF settings. We employed several analytic approaches to control for potential confounders, including multivariable analysis, instrumental variable analysis and inverse probability weighting with propensity scores. The multivariable approach

© 2019 Hong I et al. *JAMA Network Open.*

used ordinary least squares adjusting for covariates. Next, we employed inverse probability treatment weighting with propensity scores with and without multilevel adjustment.

The propensity score was generated with a logistic regression model using average treatment effect estimation that incorporated all covariates listed in eTables 4 and 5.[9] To assess the balance of covariates, we report *p*-values from Chi-square tests with propensity scores weighting. Average treatment was estimated with the weight using the inverse probability of receiving the care delivered to patients. A normalized weight was used to avoid extreme propensity scores by dividing each individual propensity score by the mean of all propensity scores. Next, we used hierarchical general linear mixed models to account for patients nested within hospitals. In these models, we used inverse probability treatment weighting with propensity scores adjusted for the patient and hospital characteristics which were not balanced after propensity score weighting. Additionally, we used ordinary least squares models with inverse probability treatment weighting with propensity scores also adjusted for unbalanced covariates, to compare functional status outcomes (mobility and self-care) at discharge between IRFs and SNFs.

As a complement to the ordinary least squares and hierarchical general linear mixed modeling, we employed instrumental variable analysis to adjust for unmeasured confounders across patients and facilities.[10] The instrumental variables included: (1) the difference between the distance from the acute hospital to the nearest IRF and the distance from the acute hospital to the nearest SNF; (2) the difference between the distance from the beneficiary's residence to the nearest IRF and the distance from the beneficiary's residence to the nearest SNF; (3) the number of stroke patients in the hospital referral region in 2013-2014 who are discharged to an IRF, divided by the number discharged to an IRF or SNF, expressed as a percentage; and (4) the previous discharge location assignment (IRF or SNF) for patients with the same type of stroke from the same acute hospital (eTable 7). The third and fourth instrumental variables were determined using the 142,542 stroke discharges selected during the study sample selection (step 7, eFigure 1). For the fourth instrumental variable, in the case of a tie between the previous discharges to IRFs and SNFs, a missing value was assigned for the index stroke discharge. Also, for a given hospital and a given stroke type, the first index discharge in the study period would have a missing value for this variable. Therefore, 91,016 records with non-missing data remained for this instrumental variable analysis. Prior to the analyses, for each instrumental variable, we examined the balance in covariates' distribution using standardized difference. For the first three instrumental variables, we created binary variables based on the median and investigated the balance between the two groups. The standardized differences were calculated using the SAS macro %stddiff[11] and are presented in eTable 8. A difference of less than 0.1 (or 10%) is generally considered a small difference between two groups.[12] Instrumental variable modeling was done using R's ivmodel package,[13] treating as endogenous the same adjustment variables used in propensity modeling, and reporting the two-stage least square estimates.

Partial F tests were used for the null hypothesis of no association between each instrument and SNF versus IRF care. To evaluate the strength of instrumental variables, we considered the first-stage F statistic for both mobility and self-care outcomes. The lowest F statistics were for the differential distance between the beneficiary and the nearest IRF or SNF (F=642.9), followed by differential distance between the acute hospital and the nearest IRF or SNF (F=2105.4), previous IRF or SNF assignment from the acute hospital by stroke type (F=2501.2) and percentage of IRF patients within the hospital referral region (F=5032.6). In other words, all F statistics were greater than 630. An F statistic greater than 10 suggests that an instrument is not weak. We utilized the Hausman test to examine if any variables were correlated with the residuals in the ordinary least square regression which produce biased estimates. In such a situation, the need for the instrumental variable approach is supported.[14] All test results were significant (p<0.0001), indicating bias of the ordinary least square approach and justifying the instrumental variable modeling approach. Besides the previous IRF or SNF assignment, the other three instrumental variables were used as continuous measures in the instrumental variable analyses. We estimated the parameters using 2-stage least square regression. For the control outcome of 30- to 365-day mortality, the parameters were estimated from 2-stage residual inclusion models because the outcome was dichotomous.

© 2019 Hong I et al. *JAMA Network Open.*

eTable 3. Comparison of Inpatient Rehabilitation Facility-Patient Assessment Instrument (IRF-PAI) With Minimum Data Set 3.0 (MDS) Items in the Mobility and Self-care Construct

Construct	IRF-PAI	MDS
**Mobility**	Transfer (Bed, Chair, Wheelchair)	Bed mobility
	Transfer (Toilet)	Transfer
	Transfer (Tub, Shower)	—
	Locomotion (Walk/Wheelchair)	Walk in room
	—	Walk in corridor
	—	Locomotion on unit
	—	Locomotion off unit
	Locomotion (Stairs)	—
**Self-care**	Eating	Eating
	Grooming	Personal hygiene
	Bathing	Bathing
	Dressing_Upper	Dressing
	Dressing_Lower	—
	Toileting	Toilet use

© 2019 Hong I et al. *JAMA Network Open.*

eTable 4. Demographics Across Inpatient Rehabilitation Facilities (IRFs) and Skilled Nursing Facilities (SNFs) Before and After Inverse Probability of Treatment Weighting

	Demographic Distributions before IPTW			Demographic Distributions after IPTW		
**Variable**	**IRF (N=66082), No. (%)**	**SNF (N=33103), No. (%)**	***p*†**	**IRF (N=66074, weighted N=49395), No. (%)**	**SNF (N=33052, weighted N=49731), No. (%)**	***p*†**
**Age (years)**						0.9899
66-69	7959 (12.04)	1869 (5.65)	<.0001*	4917 (9.95)	4900 (9.85)	
70-74	11994 (18.15)	3244 (9.80)		7614 (15.42)	7666 (15.42)	
75-79	13421 (20.31)	4931 (14.90)		9181 (18.59)	9201 (18.50)	
80-84	13931 (21.08)	6978 (21.08)		10419 (21.09)	10555 (21.23)	
85+	18777 (28.41)	16081 (48.58)		17262 (34.95)	17406 (35.00)	
**Sex**						0.6772
Male	29620 (44.82)	11637 (35.15)	<.0001*	20535 (41.57)	20761 (41.75)	
Female	36462 (55.18)	21466 (64.85)		28860 (58.43)	28970 (58.25)	
**Race**						0.8917
Non-Hispanic White	52826 (79.94)	26775 (80.88)	<.0001*	39685 (80.34)	40040 (80.51)	
Non-Hispanic Black	7753 (11.73)	3915 (11.85)		5819 (11.78)	5862 (11.79)	
Hispanic	3202 (4.85)	1371 (4.14)		2246 (4.55)	2210 (4.44)	
Other race	2301 (3.48)	1042 (3.15)		1643 (3.33)	1618 (3.25)	
**Stroke type**						0.9083
Ischemic	58872 (89.09)	29272 (88.43)	.0018*	43901 (88.88)	44214 (88.91)	
Hemorrhagic	7210 (10.91)	3831 (11.57)		5494 (11.12)	5517 (11.09)	
**Length of stay in acute care**						0.6475
1-3	28099 (42.52)	9723 (29.37)	<.0001*	18780 (38.02)	18626 (37.45)	
4-7	29996 (45.39)	16403 (49.55)		23145 (46.86)	23521 (47.30)	
8-11	5839 (8.84)	4390 (13.26)		5075 (10.28)	5182 (10.42)	
12-25	2066 (3.13)	2403 (7.26)		2269 (4.59)	2271 (4.57)	
26+	82 (0.12)	184 (0.56)		125 (0.25)	129 (0.26)	
**Admission function score‡**						
[a] Mobility, Mean (SD)	44.2 (7.4)	40.8 (9.4)	<.0001*	43.3 (6.6)	43.7 (12.0)	<.0001*
[b] Self-Care, Mean (SD)	45.0 (11.1)	41.9 (11.7)	<.0001*	44.0 (9.8)	44.3 (14.3)	0.0012*
**Medicaid eligibility (Yes)**	10454 (15.82)	7222 (21.82)	<.0001*	8844 (17.91)	8991 (18.08)	0.5632
**Stay in ICU/CCU (Yes)**	39195 (59.31)	17178 (51.89)	<.0001*	28165 (57.02)	28463 (57.24)	0.5957
**Urban hospital (Yes)**	60114 (90.97)	28207 (85.21)	<.0001*	44016 (89.11)	44337 (89.15)	0.8605
**Hospital control type**						0.9054
Profit	9480 (14.35)	4074 (12.31)	<.0001*	6774 (13.72)	6865 (13.81)	
Non Profit	48815 (73.87)	24848 (75.06)		36639 (74.18)	36807 (74.01)	
Other	7787 (11.78)	4181 (12.63)		5980 (12.11)	6057 (12.18)	
**Swing bed (Yes)**	1710 (2.59)	2023 (6.11)	<.0001*	1858 (3.76)	1871 (3.76)	0.9971
**Rehabilitation unit in IRF (Yes)**	40742 (61.65)	14657 (44.28)	<.0001*	27591 (55.86)	27624 (55.55)	0.4394
**Teaching hospital (Yes)**	34919 (52.84)	15858 (47.91)	<.0001*	25248 (51.12)	25576 (51.43)	0.4479
**Number of stroke Discharges, Mean (SD)‡**	248.0 (175.9)	218.7 (174.8)	<.0001*	238.9 (149.0)	240.7 (226.4)	0.2501
**Number of beds in the hospital, Mean (SD)‡**	463.0 (329.2)	414.2 (332.0)	<.0001*	447.1 (278.5)	448.9 (427.8)	0.5563

Admission function scores for Mobility and Self-Care were scaled on 0-100 point scales.

© 2019 Hong I et al. *JAMA Network Open.*

[a], IRF-PAI = Transfer (Bed, Chair, Wheelchair) + Transfer (Toilet) + Transfer (Tub, Shower) + Locomotion (Walk/Wheelchair) + Locomotion (Stairs)
MDS = Bed mobility + Transfer + Walk in room + Walk in corridor + Locomotion on unit + Locomotion off unit
[b], IRF-PAI = Eating + Grooming + Bathing + Dressing_Upper + Dressing_Lower + Toileting
MDS = Dressing + Eating + Toilet use + Personal hygiene + Bathing
†, Chi-square test
‡, Wilcoxon Rank Sum test
*, Statistically significant at a significance level of 0.05
IPTW: inverse probability of treatment weighting; ICU: intensive care unit; CCU: cardiac care unit; SD: standard deviation.

© 2019 Hong I et al. *JAMA Network Open.*

eTable 5. Stroke Comorbidities Across Inpatient Rehabilitation Facilities (IRFs) and Skilled Nursing Facilities (SNFs) Before and After Inverse Probability of Treatment Weighting

	**Demographic Distributions before IPTW**			**Demographic Distributions after IPTW**		
**Comorbidities**	**IRF (N=66082), No. (%)**	**SNF (N=33103), No. (%)**	***p*†**	**IRF (N=66074, weighted N=49395), No. (%)**	**SNF (N=33052, weighted N=49731), No. (%)**	***p*†**
Hemiplegia/Hemiparesis	31387 (47.50)	12154 (36.72)	<.0001*	21575 (43.68)	21255 (42.74)	0.0236*
Specified Heart Arrhythmias	22701 (34.35)	13133 (39.67)	<.0001*	17975 (36.39)	18212 (36.62)	0.5570
Diabetes without Complication	19860 (30.05)	9169 (27.70)	<.0001*	14548 (29.45)	14682 (29.52)	0.8534
Congestive Heart Failure	14308 (21.65)	8918 (26.94)	<.0001*	11623 (23.53)	11762 (23.65)	0.7204
Acute Renal Failure	9320 (14.10)	5833 (17.62)	<.0001*	7620 (15.43)	7815 (15.72)	0.3218
Chronic Obstructive Pulmonary Disease	9585 (14.50)	5317 (16.06)	<.0001*	7529 (15.24)	7755 (15.60)	0.2313
Vascular Disease	8332 (12.61)	4242 (12.81)	0.3579	6326 (12.81)	6429 (12.93)	0.6601
Ischemic or Unspecified Stroke	5333 (8.07)	2807 (8.48)	0.0268*	4118 (8.34)	4274 (8.60)	0.2653
Diabetes with Chronic Complications	5006 (7.58)	2388 (7.21)	0.0409*	3749 (7.59)	3908 (7.86)	0.2353
Coagulation Defects and Other Specified Hematological Disorders	4081 (6.18)	2196 (6.63)	0.0052*	3161 (6.40)	3206 (6.45)	0.8113
Seizure Disorders and Convulsions	3557 (5.38)	2383 (7.20)	<.0001*	2975 (6.02)	2974 (5.98)	0.8182
Coma, Brain Compression/Anoxic Damage	3802 (5.75)	1851 (5.59)	0.2999	2783 (5.63)	2757 (5.54)	0.6243
Cardio-Respiratory Failure and Shock	3230 (4.89)	2016 (6.09)	<.0001*	2668 (5.40)	2716 (5.46)	0.7415
Cerebral Hemorrhage	3211 (4.86)	1600 (4.83)	0.8589	2389 (4.84)	2428 (4.88)	0.7969
Protein-Calorie Malnutrition	2506 (3.79)	2176 (6.57)	<.0001*	2337 (4.73)	2414 (4.85)	0.4588
Rheumatoid Arthritis and Inflammatory Connective Tissue Disease	2601 (3.74)	1392 (4.21)	0.0421*	1995 (4.04)	2070 (4.16)	0.4420
Morbid Obesity	2629 (3.98)	1141 (3.45)	<.0001*	1895 (3.84)	1891 (3.80)	0.8305
Aspiration and Specified Bacterial Pneumonias	2204 (3.34)	1536 (4.64)	<.0001*	1856 (3.76)	1875 (3.77)	0.9224
Acute Myocardial Infarction	2144 (3.24)	1464 (4.42)	<.0001*	1803 (3.65)	1814 (3.65)	0.9840
Other Significant Endocrine and Metabolic Disorders	2271 (3.44)	1199 (3.62)	0.1340	1760 (3.56)	1754 (3.53)	0.8017
Septicemia, Sepsis, Systemic Inflammatory Response Syndrome/Shock	1578 (2.39)	1288 (3.89)	<.0001*	1444 (2.92)	1460 (2.94)	0.9232
Parkinson's and Huntington's Diseases	1466 (2.22)	1025 (3.10)	<.0001*	1241 (2.51)	1244 (2.50)	0.9266
Breast, Prostate, and Other Cancers and Tumors	1581 (2.39)	856 (2.59)	0.0636	1200 (2.43)	1211 (2.44)	0.9592
Major Depressive, Bipolar, and Paranoid Disorders	1117 (1.69)	800 (2.42)	<.0001*	968 (1.96)	1018 (2.05)	0.4381

© 2019 Hong I et al. *JAMA Network Open.*

Vascular Disease with Complications	990 (1.50)	582 (1.76)	0.0020*	794 (1.61)	783 (1.57)	0.7420
Unstable Angina and Other Acute Ischemic Heart Disease	913 (1.38)	536 (1.62)	0.0033*	732 (1.48)	728 (1.47)	0.8494
Intestinal Obstruction/Perforation	832 (1.26)	585 (1.77)	<.0001*	714 (1.45)	741 (1.49)	0.6393
Hip Fracture/Dislocation	681 (1.03)	662 (2.00)	<.0001*	674 (1.36)	686 (1.38)	0.8654
Metastatic Cancer and Acute Leukemia	774 (1.17)	457 (1.38)	0.0050*	637 (1.29)	634 (1.28)	0.8771
Vertebral Fractures without Spinal Cords Injury	637 (0.96)	549 (1.66)	<.0001*	590 (1.19)	596 (1.20)	0.9494
**Cognitive Function related Variables**						
Dementia without Complication	8903 (13.47)	9910 (29.94)	<.0001*	6085 (12.32)	6172 (12.41)	0.7137
Delirium and Encephalopathy	6339 (9.59)	5736 (17.33)	<.0001*	9366 (18.96)	9498 (19.10)	0.6408
Nonpsychotic Organic Brain Syndromes/Conditions	2046 (3.10)	1322 (3.99)	<.0001*	1710 (3.46)	1767 (3.55)	0.5324
Dementia with Complications	1123 (1.70)	1363 (4.12)	<.0001*	1224 (2.48)	1244 (2.50)	0.8393
Concussion/Head Injury	858 (1.30)	696 (2.10)	<.0001*	782 (1.58)	824 (1.66)	0.4570
Schizophrenia	199 (0.30)	187 (0.56)	<.0001*	198 (0.40)	202 (0.41)	0.9154

†, Chi-square test; *, Statistically significant at a significance level of 0.05.
IPTW: inverse probability of treatment weighting.

© 2019 Hong I et al. *JAMA Network Open.*

eTable 6. Additional Diagnoses Related to Cognitive Function

Diagnosis	ICD-9 Codes
Delirium and Encephalopathy	293.0, 293.1, 293.81, 293.82, 293.83, 293.84, 293.89, 293.9, 323.71, 348.30, 348.31, 348.39, 349.82, 368.16, 780.1
Dementia With Complications	290.11, 290.12, 290.13, 290.20, 290.21, 290.3, 290.41, 290.42, 290.43, 294.11, 294.21, 331.3, 331.4, 331.5
Dementia Without Complication	046.0, 046.11, 046.19, 046.2, 046.3, 046.71, 046.72, 046.79, 046.8, 046.9, 290.0, 290.10, 290.40, 290.8, 290.9, 294.0, 294.10, 294.20, 294.8, 294.9, 330.0, 330.1, 330.2, 330.3, 330.8, 330.9, 331.0, 331.11, 331.19, 331.2, 331.6, 331.7, 331.81, 331.82, 331.89, 331.9
Nonpsychotic Organic Brain Syndromes/Conditions	310.0, 310.1, 310.2, 310.81, 310.89, 310.9, 331.83, 348.0, 348.2, 348.81, 348.89, 348.9, 349.2, 349.39, 349.81, 349.89, 349.9, 797
Schizophrenia	295.00, 295.01, 295.02, 295.03, 295.04, 295.05, 295.10, 295.11, 295.12, 295.13, 295.14, 295.15, 295.20, 295.21, 295.22, 295.23, 295.24, 295.25, 295.30, 295.31, 295.32, 295.33, 295.34, 295.35, 295.40, 295.41, 295.42, 295.43, 295.44, 295.45, 295.50, 295.51, 295.52, 295.53, 295.54, 295.55, 295.60, 295.61, 295.62, 295.63, 295.64, 295.65, 295.70, 295.71, 295.72, 295.73, 295.74, 295.75, 295.80, 295.81, 295.82, 295.83, 295.84, 295.85, 295.90, 295.91, 295.92, 295.93, 295.94, 295.95
Severe Head Injury	800.03, 800.04, 800.05, 800.13, 800.14, 800.15, 800.23, 800.24, 800.25, 800.33, 800.34, 800.35, 800.43, 800.44, 800.45, 800.53, 800.54, 800.55, 800.63, 800.64, 800.65, 800.73, 800.74, 800.75, 800.83, 800.84, 800.85, 800.93, 800.94, 800.95, 801.03, 801.04, 801.05, 801.13, 801.14, 801.15, 801.23, 801.24, 801.25, 801.33, 801.34, 801.35, 801.43, 801.44, 801.45, 801.53, 801.54, 801.55, 801.63, 801.64, 801.65, 801.73, 801.74, 801.75, 801.83, 801.84, 801.85, 801.93, 801.94, 801.95, 803.03, 803.04, 803.05, 803.13, 803.14, 803.15, 803.23, 803.24, 803.25, 803.33, 803.34, 803.35, 803.43, 803.44, 803.45, 803.53, 803.54, 803.55, 803.63, 803.64, 803.65, 803.73, 803.74, 803.75, 803.83, 803.84, 803.85, 803.93, 803.94, 803.95, 804.03, 804.04, 804.05, 804.13, 804.14, 804.15, 804.23, 804.24, 804.25, 804.33, 804.34, 804.35, 804.43, 804.44, 804.45, 804.53, 804.54, 804.55, 804.63, 804.64, 804.65, 804.73, 804.74, 804.75, 804.83, 804.84, 804.85, 804.93, 804.94, 804.95, 851.03, 851.04, 851.05, 851.13, 851.14, 851.15, 851.23, 851.24, 851.25, 851.33, 851.34, 851.35, 851.43, 851.44, 851.45, 851.53, 851.54, 851.55, 851.63, 851.64, 851.65, 851.73, 851.74, 851.75, 851.83, 851.84, 851.85, 851.93, 851.94, 851.95, 852.03, 852.04, 852.05, 852.13, 852.14, 852.15, 852.23, 852.24, 852.25, 852.33, 852.34, 852.35, 852.43, 852.44, 852.45, 852.53, 852.54, 852.55, 853.03, 853.04, 853.05, 853.13, 853.14, 853.15, 854.03, 854.04, 854.05, 854.13, 854.14, 854.15, 800.00, 800.01, 800.02, 800.06, 800.09, 800.10, 800.11, 800.12, 800.16, 800.19, 800.20, 800.21, 800.22, 800.26, 800.29, 800.30, 800.31, 800.32, 800.36, 800.39, 800.40, 800.41, 800.42, 800.46, 800.49, 800.50, 800.51, 800.52, 800.56, 800.59, 800.60, 800.61, 800.62, 800.66, 800.69, 800.70, 800.71, 800.72, 800.76, 800.79, 800.80, 800.81, 800.82, 800.86, 800.89, 800.90, 800.91, 800.92, 800.96, 800.99, 801.00, 801.01, 801.02, 801.06, 801.09, 801.10, 801.11, 801.12, 801.16, 801.19, 801.20, 801.21, 801.22, 801.26, 801.29, 801.30, 801.31, 801.32, 801.36, 801.39, 801.40, 801.41, 801.42, 801.46, 801.49, 801.50, 801.51, 801.52, 801.56, 801.59, 801.60, 801.61, 801.62, 801.66, 801.69, 801.70, 801.71, 801.72, 801.76, 801.79, 801.80, 801.81, 801.82, 801.86, 801.89, 801.90, 801.91, 801.92, 801.96, 801.99,

© 2019 Hong I et al. *JAMA Network Open.*

	802.20, 802.21, 802.22, 802.23, 802.24, 802.25, 802.26, 802.27, 802.28, 802.29, 802.30, 802.31, 802.32, 802.33, 802.34, 802.35, 802.36, 802.37, 802.38, 802.39, 802.4, 802.5, 802.6, 802.7, 802.8, 802.9, 803.00, 803.01, 803.02, 803.06, 803.09, 803.10, 803.11, 803.12, 803.16, 803.19, 803.20, 803.21, 803.22, 803.26, 803.29, 803.30, 803.31, 803.32, 803.36, 803.39, 803.40, 803.41, 803.42, 803.46, 803.49, 803.50, 803.51, 803.52, 803.56, 803.59, 803.60, 803.61, 803.62, 803.66, 803.69, 803.70, 803.71, 803.72, 803.76, 803.79, 803.80, 803.81, 803.82, 803.86, 803.89, 803.90, 803.91, 803.92, 803.96, 803.99, 804.00, 804.01, 804.02, 804.06, 804.09, 804.10, 804.11, 804.12, 804.16, 804.19, 804.20, 804.21, 804.22, 804.26, 804.29, 804.30, 804.31, 804.32, 804.36, 804.39, 804.40, 804.41, 804.42, 804.46, 804.49, 804.50, 804.51, 804.52, 804.56, 804.59, 804.60, 804.61, 804.62, 804.66, 804.69, 804.70, 804.71, 804.72, 804.76, 804.79, 804.80, 804.81, 804.82, 804.86, 804.89, 804.90, 804.91, 804.92, 804.96, 804.99, 850.2, 850.3, 850.4, 851.00, 851.01, 851.02, 851.06, 851.09, 851.10, 851.11, 851.12, 851.16, 851.19, 851.20, 851.21, 851.22, 851.26, 851.29, 851.30, 851.31, 851.32, 851.36, 851.39, 851.40, 851.41, 851.42, 851.46, 851.49, 851.50, 851.51, 851.52, 851.56, 851.59, 851.60, 851.61, 851.62, 851.66, 851.69, 851.70, 851.71, 851.72, 851.76, 851.79, 851.80, 851.81, 851.82, 851.86, 851.89, 851.90, 851.91, 851.92, 851.96, 851.99, 852.00, 852.01, 852.02, 852.06, 852.09, 852.10, 852.11, 852.12, 852.16, 852.19, 852.20, 852.21, 852.22, 852.26, 852.29, 852.30, 852.31, 852.32, 852.36, 852.39, 852.40, 852.41, 852.42, 852.46, 852.49, 852.50, 852.51, 852.52, 852.56, 852.59, 853.00, 853.01, 853.02, 853.06, 853.09, 853.10, 853.11, 853.12, 853.16, 853.19, 854.00, 854.01, 854.02, 854.06, 854.09, 854.10, 854.11, 854.12, 854.16, 854.19, 905.0, 907.0
Concussion or Unspecified Head Injury	850.0, 850.11, 850.12, 850.5, 850.9, 959.01

ICD-9: International Classification of Diseases, Ninth Revision.

© 2019 Hong I et al. *JAMA Network Open.*

eTable 7. Instrumental Variables Across Inpatient Rehabilitation Facilities (IRFs) and Skilled Nursing Facilities (SNFs)

**Instrumental Variable**	**IRF (N=66082), No. (%)**	**SNF (N=33103), No. (%)**	***p***
**Differential distance from acute to nearest IRF/SNF, Mean (SD)**	2.7 (9.2)	8.5 (15.8)	<.0001*
**Differential distance from beneficiary to nearest IRF/SNF, Mean (SD)**	8.9 (14.9)	12.4 (16.6)	<.0001*
**Percentage of IRFs with the acute hospital HRR, Mean (SD)**	53.3 (12.5)	45.7 (13.3)	<.0001*
**Previous IRF/SNF assignment by stroke type within each hospital**			
IRF	37843 (62.17)	11674 (38.76)	<.0001*
SNF	23026 (37.83)	18448 (61.24)	

*, Statistically significant at a significance level of 0.05.
SD: standard deviation; HRR: hospital referral region.

© 2019 Hong I et al. *JAMA Network Open.*

eTable 8. Standardized Difference for Instrumental Variables

		Differential Distance Between Acute to Nearest IRF/SNF (N=99,185)		Differential Distance Between the beneficiaries to Nearest IRF/SNF (N=99,185)		% IRF Patient within each HRR (N=99,185)		Previous IRF/SNF Assigned by Stroke Type (N=91,016)	
**Variables**	**Standardized Difference IRF/SNF**	**Standardized Difference**	**Balanced**	**Standardized Difference**	**Balanced**	**Standardized Difference**	**Balanced**	**Standardized Difference**	**Balanced**
Age	0.481	0.091		0.053		0.037		0.074	
Sex	-0.198	0.025		-0.027		-0.019		-0.013	
Race	0.040	0.048		0.252	N	0.147	N	0.041	
Stroke Type	0.021	0.072		-0.010		0.014		0.036	
Length of Stay in Acute Care	0.333	0.044		0.031		0.080		0.082	
Admission Mobility Score	0.396	-0.106	N	-0.030		0.053		0.090	
Admission Self-Care Score	0.277	-0.111	N	-0.024		-0.021		0.036	
Medicaid Eligibility	-0.154	0.015		-0.038		0.017		-0.023	
Stay in ICU/CCU	0.150	-0.083		-0.100		0.098		0.068	
Urban Hospital	0.179	-0.232	N	-0.312	N	0.141	N	0.181	N
Hospital Control Type	0.063	0.057		0.097		0.155	N	0.076	
Swing Bed	-0.173	0.312	N	0.236	N	-0.042		-0.162	N
Rehab Unit IRF	0.354	-1.726	N	-0.389	N	0.154	N	0.297	N
Teaching Hospital	0.099	-0.330	N	-0.144	N	0.019		0.072	
Number of Stroke Discharge	0.167	-0.606	N	-0.069		-0.035		0.128	N
Count of Beds in the Hospital	0.148	-0.618	N	-0.196	N	0.010		0.111	N
Hemiplegia/Hemiparesis	0.220	-0.125	N	-0.031		0.015		0.036	
Specified Heart Arrhythmias	-0.110	0.004		-0.001		-0.023		-0.016	
Diabetes without Complication	0.052	-0.012		-0.010		0.023		0.015	
Congestive Heart Failure	-0.124	-0.016		0.002		-0.015		-0.004	

© 2019 Hong I et al. *JAMA Network Open.*

Acute Renal Failure	-0.096	-0.010		-0.025		0.003		0.005	
Chronic Obstructive Pulmonary Disease	-0.043	-0.001		0.027		-0.011		-0.001	
Vascular Disease	-0.006	-0.018		0.000		-0.003		0.004	
Ischemic or Unspecified Stroke	-0.015	-0.002		0.000		0.005		0.008	
Diabetes with Chronic Complications	0.014	-0.019		-0.027		0.022		0.022	
Coagulation Defects and Other Specified Hematological Disorders	-0.019	-0.009		-0.015		-0.008		0.002	
Seizure Disorders and Convulsions	-0.075	-0.013		-0.013		0.014		0.000	
Coma, Brain Compression/Anoxic Damage	0.007	-0.039		0.003		-0.020		-0.021	
Cardio-Respiratory Failure and Shock	-0.053	-0.026		0.003		-0.014		0.003	
Cerebral Hemorrhage	0.001	-0.038		-0.009		-0.003		0.006	
Protein-Calorie Malnutrition	-0.126	-0.026		-0.019		0.007		0.007	
Rheumatoid Arthritis and Inflammatory Connective Tissue Disease	-0.014	-0.005		0.008		0.000		0.006	
Morbid Obesity	0.028	-0.026		0.013		0.011		0.016	
Aspiration and Specified Bacterial Pneumonias	-0.067	0.005		0.003		-0.013		-0.008	
Acute Myocardial Infarction	-0.061	0.011		0.010		0.001		-0.002	
Other Significant Endocrine and Metabolic Disorders	-0.010	-0.018		-0.007		-0.006		-0.003	
Septicemia, Sepsis, Systemic Inflammatory	-0.086	-0.014		-0.013		-0.018		-0.011	

© 2019 Hong I et al. *JAMA Network Open.*

Response Syndrome/Shock									
Parkinson's and Huntington's Diseases	-0.055	0.004		-0.014		0.021		-0.007	
Breast, Prostate, and Other Cancers and Tumors	-0.012	-0.001		-0.014		-0.006		-0.006	
Major Depressive, Bipolar, and Paranoid Disorders	-0.051	-0.002		-0.021		0.017		-0.004	
Vascular Disease with Complications	-0.021	-0.003		-0.005		-0.016		-0.011	
Unstable Angina and Other Acute Ischemic Heart Disease	-0.020	0.021		-0.010		0.019		0.000	
Intestinal Obstruction/Perforation	-0.042	-0.007		-0.012		-0.001		-0.002	
Hip Fracture/Dislocation	-0.079	0.012		-0.006		0.006		-0.009	
Metastatic Cancer and Acute Leukemia	-0.019	0.001		-0.019		0.012		0.007	
Vertebral Fractures without Spinal Cords Injury	-0.061	0.019		-0.007		0.004		-0.004	
**Cognitive Function related Variables**									
Dementia with Complication	-0.144	0.014		-0.007		0.001		-0.018	
Delirium and Encephalopathy	-0.228	-0.014		-0.021		-0.021		-0.004	
Nonpsychotic Organic Brain Syndromes/Conditions	-0.049	-0.016		-0.022		-0.007		0.006	
Dementia without Complications	-0.408	0.031		-0.034		-0.026		-0.040	
Concussion/Head Injury	-0.062	-0.001		-0.018		0.002		0.005	

© 2019 Hong I et al. *JAMA Network Open.*

Schizophrenia	-0.040	0.013		-0.016		-0.003		-0.013	

An imbalance was determined when the standardized difference was greater than 0.1 or smaller than -0.1.
IRF: inpatient rehabilitation facility; SNF: skilled nursing facility; HRR: hospital referral region; ICU: intensive care unit; CCU: cardiac care unit.

© 2019 Hong I et al. *JAMA Network Open.*

eTable 9. E-values for Mobility and Self-care Scores and 30- to 365-Day Mortality From Hospital Discharge

Change in mobility and self-care scores from admission to discharge

**Outcome**	***d*, standardized difference (IRF vs. SNF)**	**SE**	**RR ≈ $e^{0.91 \times d}$**	**RR LCL ≈ $e^{0.91 \times d - 1.78 \times se}$**	**RR UCL ≈ $e^{0.91 \times d + 1.78 \times se}$**	**E-Value, point estimate**	**E-Value, CI [a]**	**Interpretation**
**Mobility**	0.924	0.006	2.319	2.294	2.345	4.068	4.016	An unmeasured confounder associated with mobility score change from admission to discharge and discharge destination (IRF vs SNF) by a risk ratio of **4**-fold each could explain away the lower confidence limit, but weaker confounding could not.
**Self-Care**	0.974	0.006	2.427	2.401	2.453	4.288	4.234	An unmeasured confounder associated with self-care score change from admission to discharge and discharge destination (IRF vs SNF) by a risk ratio of **4.2**-fold each could explain away the lower confidence limit, but weaker confounding could not.

30- to 365-day mortality from hospital discharge

**OR (IRF vs. SNF)**	**OR, 95% CI**	**RR ≈ √OR**	**RR Lower CL**	**RR Upper CL**	**E-Value, point estimate**	**E-Value, CI [a]**	**Interpretation**
0.72	0.69, 0.74	0.85	0.83	0.86	1.64	1.60	An unmeasured confounder associated with death and discharge destination (IRF vs SNF) by a risk ratio of **1.6**-fold each could explain away the upper confidence limit, but weaker confounding could not.

© 2019 Hong I et al. *JAMA Network Open.*

[a], It is only meaningful to calculate the E-value for the limit of the confidence interval closest to the null (RR=1).
IRF, inpatient rehabilitation facility; SNF, skilled nursing facility; SE, standard error; OR, odds ratio; CI, confidence interval; LCL, lower confidence limit; ULC, upper confidence limit; RR, relative risk.

© 2019 Hong I et al. *JAMA Network Open.*

eReferences

1. Centers for Medicare and Medicaid Services. The Inpatient Rehabilitation Facility–Patient Assessment Instrument (IRF-PAI) Training Manual. Modified July 31, 2018. https://www.cms.gov/Medicare/Medicare-Fee-for-Service-Payment/InpatientRehabFacPPS/IRFPAI.html. Accessed May 10, 2019.
2. Centers for Medicare and Medicaid Services. Assessments for the Resident Assessment Instrument (RAI). In Minimum Data Set, Version 3.0 (MDS 3.0) RAI Manual. https://www.cms.gov/Medicare/Quality-Initiatives-Patient-Assessment-Instruments/NursingHomeQualityInits/MDS30RAIManual.html. Accessed May 10, 2019.
3. Mallinson TR, Bateman J, Tseng HY, et al. A comparison of discharge functional status after rehabilitation in skilled nursing, home health, and medical rehabilitation settings for patients after lower-extremity joint replacement surgery. *Arch Phys Med Rehabil.* 2011;92(5):712-720.
4. Masters GN. Common-person equating with the Rasch model. *Applied Psychological Measurement.* 1985;9(1):73-82.
5. Velozo CA, Byers KL, Wang YC, Joseph BR. Translating measures across the continuum of care: using Rasch analysis to create a crosswalk between the Functional Independence Measure and the Minimum Data Set. *J Rehabil Res Dev.* 2007;44(3):467-478.
6. Hong I, Woo HS, Shim S, Li CY, Yoonjeong L, Velozo CA. Equating activities of daily living outcome measures: the Functional Independence Measure and the Korean version of Modified Barthel Index. *Disabil Rehabil.* 2018;40(2):217-224.
7. Pope GC, Kautter J, Ellis RP, et al. Risk adjustment of Medicare capitation payments using the CMS-HCC model. *Health Care Financing Review*. 2004;25(4):119.
8. Hong I, Karmarkar A, Chan W, et al. Discharge patterns for ischemic and hemorrhagic stroke patients going from acute care hospitals to inpatient and skilled nursing rehabilitation. *Am J Phys Med Rehabil.* 2018;97(9):636-645.
9. Rosenbaum PR, Rubin DB. The central role of the propensity score in observational studies for causal effects. *Biometrika.* 1983;70(1):41-55.
10. Kuo Y-F, Chen N-W, Baillargeon J, Raji MA, Goodwin JS. Potentially preventable hospitalizations in medicare patients with diabetes: a comparison of primary care provided by nurse practitioners versus physicians. *Med Care.* 2015;53(9):776-783.
11. Yang D, Dalton JE. A unified approach to measuring the effect size between two groups using SAS®. *SAS Global Forum*. 2012;335:1-6.
12. Rubin DB. Using propensity scores to help design observational studies: application to the tobacco litigation. *Health Services and Outcomes Research Methodology*. 2001;2(3-4):169-188.
13. Jiang Y, Kang H, Small D, Zhao Q. ivmodel: Statistical inference and sensitivity analysis for instrumental variables model. R package version. 2017;1(1).
14. Hausman JA. Specification tests in econometrics. *Econometrica.* 1978;46(6):1251-1271.

© 2019 Hong I et al. *JAMA Network Open.*
